# Automated photo-aligned liquid crystal elastomer film fabrication with a low-tech, home-built robotic workstation

**DOI:** 10.1038/s41598-022-22556-8

**Published:** 2022-10-20

**Authors:** Przemysław Grabowski, Bartosz Fabjanowicz, Magdalena Podgórska, Mikołaj Rogóż, Piotr Wasylczyk

**Affiliations:** Photonic Nanostructure Facility, Faculty of Physics, University of Warsaw, Ul. Pasteura 5, 02-093 Warsaw, Poland

**Keywords:** Liquid crystals, Polymers, Optical materials, Liquid crystals, Polymers, Liquid crystals, Polymers, Design, synthesis and processing, Engineering

## Abstract

Laboratory procedures are often considered so unique that automating them is not economically justified – time and resources invested in designing, building and calibrating the machines are unlikely to pay off. This is particularly true if cheap labour force (technicians or students) is available. Yet, with increasing availability and dropping prices of many off-the-shelf components such as motorised stages, grippers, light sources (LEDs and lasers), detectors (high resolution, fast cameras), as well as user-friendly programmable microprocessors, many of the repeatable tasks may soon be within reach of either custom-built or universal lab robots. Building on our previous work on fabrication, characterization and applications of light-responsive liquid crystal elastomers (LCEs) in micro-robotics and micro-mechanics, in this paper we present a robotic workstation that can make LCE films with arbitrary molecular orientation. Based on a commercial 3D printer, the RoboLEC (Robot for LCE fabrication) performs precision component handling, structured light illumination, liquid dispensing and UV-triggered polymerization, within a four-hour-long procedure. Thus fabricated films with patterned molecular orientation are compared to the same, but handmade, structures.

## Introduction

In both utopian and dystopian visions of the future of human labor, scientific jobs are usually considered to be on the safe side. Yet, on second thought, large swaths of research work may in fact be much easier to automate than we are ready to acknowledge^1,2^.

Many laboratory activities seem to be so unique that automating them may at first appear to be well beyond what is economically justified—designing, building, testing and calibrating the (usually one-off) laboratory equipment needed would take longer than doing the task manually; this is particularly true if cheap labor is available. Yet, with increasing availability of components such as motorized stages, grippers, light sources (LEDs and lasers covering UV, visible and NIR bands), detectors (high resolution, fast cameras), and other mechanical and optical components, as well as programmable microprocessors, many daily, mundane, repeatable tasks may soon come to be automated, either by researchers themselves or by companies delivering the necessary solutions. Apart from the increased throughput, such automatization would also eliminate the human factor which often contributes to errors in complex laboratory procedures. Robotic workstations guarantee precision timing, temperature settings and reliable control of other parameters without subjective estimation.

There are two areas where automation may come into play in research: software (data acquisition, storage and processing) and hardware (sample preparation, handling, disposal). Whereas the substantial progress made in software is beyond doubt, hardware, in principle, is considered to have a much higher entry threshold. In a typical laboratory setting—be it chemistry, biology, materials science, or solid state physics—automating even part of the workflow of sample preparation and handling would require expertise in precision mechanics, electronics, robotics, perhaps combined with optics, machine vision, haptics, etc. As a result it may prove prohibitively difficult to automate even the simplest parts of the workflow, especially if cheap labor is readily available—many technicians as well as MSc and PhD students spend most of their time performing routine, repeatable tasks, like pipetting hundreds and thousands of samples for standard measurement procedures or transferring samples from one apparatus to the next.

At the same time, many solutions and sub-systems are becoming more and more easily available at the hardware end. Precision mechanics, computer-controlled drives and actuators, sensors and cameras have entered the mass market, which has dramatically boosted availability and reduced prices.

The life sciences have already benefited from laboratory automation in processing large numbers of samples. High-throughput workstations have been demonstrated for genome screening, protein purification and drug discovery, consisting of robots preparing samples (e.g. setting up transfections), moving them around (in well plates, tubes or cell culture flasks) between liquid handlers, centrifuges, incubators and plate readers^3^. While such workstations require teams of engineers (and substantial resources) to design and set up, cheap automated sample preparation and imaging is also possible, with authors often sharing the designs openly.

A pipetting robot has been developed, combined with a modular optical system and open-source software for microscopy and image analysis^4^. In high-throughput chemistry research—searching for photocatalysts for hydrogen production from water—a computer controlled system has been demonstrated, with a mobile robotic arm navigating through the laboratory, handling samples between several pieces of equipment, interpreting the results and optimizing the output^5^. Similar systems have also been scaled down (in size and cost) to fit onto a single desktop^6^.

In a robotic platform developed for organic flow synthesis, a robotic arm assembles modular units (reactors/separators) and connects reagent lines and pumps to the setup; after the synthesis is completed, the robot removes the modules to their storage^7^. The Chemputer project, in turn, sought to develop a computer program that would translate abstract chemical procedures into instructions for modular hardware of a laboratory-scale synthesis robot. The robotic platform was set on a fixed fluidic backbone, made of pumps and tubes connected to different modules: a reaction flask, temperature controlled filtration setup, liquid–liquid separator and solvent evaporation module^8^.

Inspired by such examples, in order to explore current perspectives in material (polymer) science research automation, we designed and built a compact workstation for liquid crystal elastomer (LCE) film fabrication, which we called “RoboLEC” (Robot for LCE fabrication). LCE films have been used as light- or temperature-responsive actuators in soft, millimeter-scale robotics^9^ and micromechanics^10^. In one of the possible approaches, they are prepared by photopolymerization of liquid monomers in glass cells, where the orienting layers on the glass surface (prepared by e.g. photoalignment) determine the molecular order and orientation within the film, and thus its optical properties and photo-mechanical response^11^. The ultimate goal here is a technology that would deliver LCE films with any pre-programmed director orientation within the material volume^12^. In the case of thin films, arbitrary molecular orientation means that there may be any (position-dependent) director orientation at one film surface and a different director orientation on the other surface. Some of the automated processes realized in our device are used in the industry, in particular photoalignment and photopatterning has been introduced in fabrication of liquid crystal displays (LCDs)—see^13–16^ for a review of developments therein.

## Methods

LCE film fabrication involves several stages: preparation and precision handling of glass slides that, once glued with a spacer in between, make up the cells, structured light exposure to prepare orienting layers via photoalignment^11^, and monomer mixture polymerization by UV exposure. Some of these must be performed at controlled temperatures (typically between room temperature and 200 °C).

**Figure 1:**
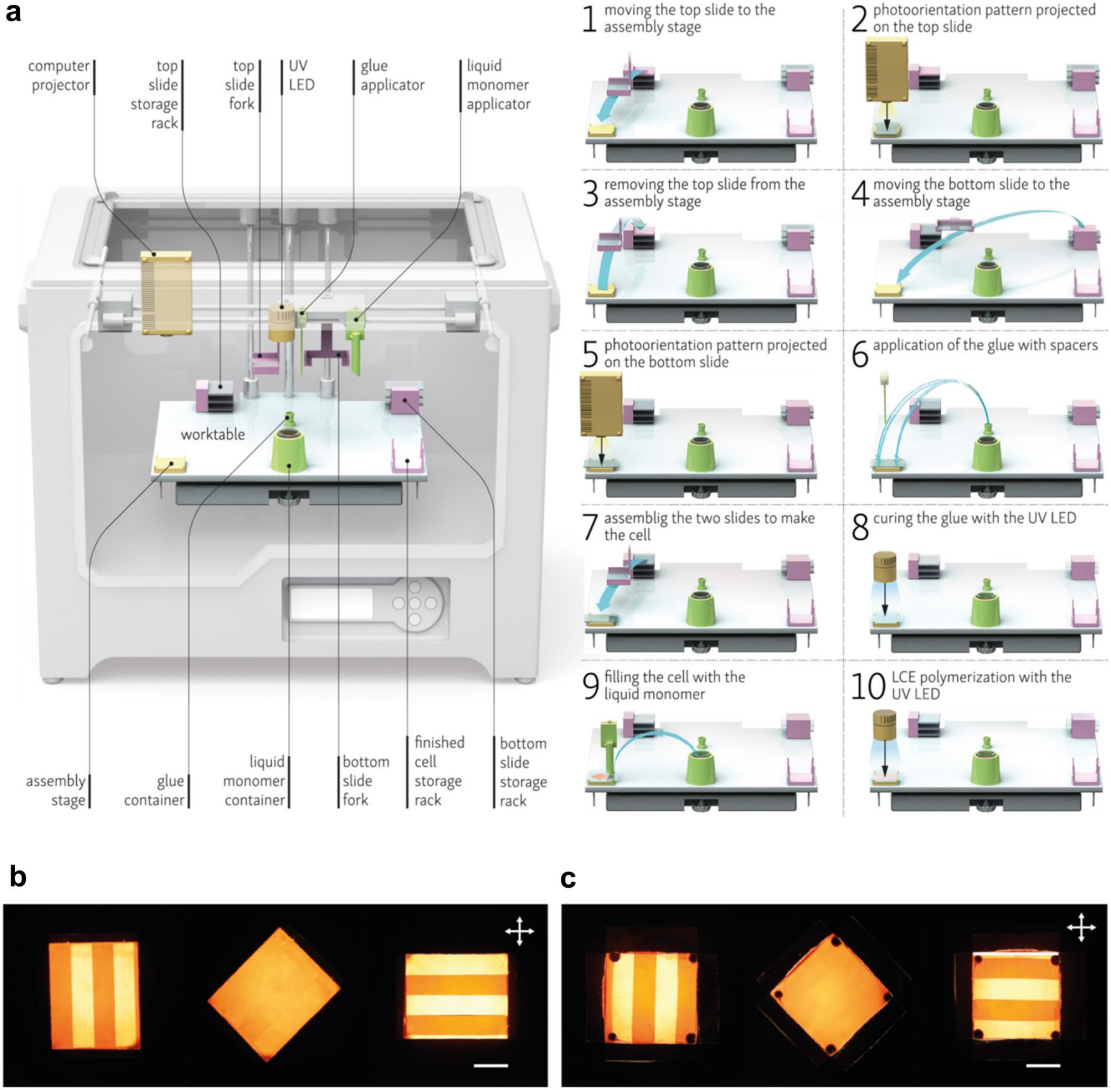
The RoboLEC robotic workstation for automated liquid crystal elastomer (LCE) film fabrication. The framework uses an extrusion 3D printer with several modifications: storage racks for the glass slides, containers for the UV glue and the LCE molten mixture, and a laser projector. Figure shows ten steps of the photoaligned LCE film fabrication (**a**), and LCE films with patterned alignment: handmade (**b**) and from the RoboLEC (**c**). Polarized white light photographs at three different orientations, the cross shows the polarizer orientation, the scale bar is 5 mm long.

The concept for our RoboLEC workstation originated from the realization that most of the steps can be straightforwardly implemented in a standard extrusion 3D printer, with minor modification to the hardware. A Flashforge Creator Pro 3D printer (227 × 148 × 150 mm^3^ workspace) was used as the platform and was connected to the internet with OctoPrint plugin for Raspberry Pi 3 to enable the G-CODE control. The extruders and fans on the printer’s head were replaced with a set of custom-made tools: two forks for glass slide handling, a glue dispenser, a heated LCE mixture applicator, and a UV (395 nm) LED for photopolymerization. Two storage racks for glass slides and one for finished cells, a stage for the alignment and assembly of cell parts, a glue container, and a heated LCE monomer container were mounted on the printer’s heated worktable. A miniature computer laser projector with a set of polarizers was installed for the photoalignment pattern generation—compare Fig. 1 and Movie 1 in the Supplementary Information. Most of the added parts were either machined from metal or 3D printed from polymers (see Table 1 in the Supplementary Information for the list of components).

## Results

RoboLEC’s process of automated LCE film fabrication, presented in ten consecutive stages in Fig. 1a, starts with lifting the first (top) glass slide (26 × 38 × 1 mm^3^, one half of a standard microscope slide) from the storage rack and placing it onto the assembly stage. The glass slides have been prepared by spin-coating with a photoalignment azopolyimide (PI) solution (∼1%) in N-methyl-2-pyrrolidone, dried, and heated at 130 °C for 2.5 h to evaporate the solvent^17^. At the assembly stage, the computer laser projector illuminates the glass coated with the photoalignment layer with arbitrary patterns through one or two perpendicular polarizers (the blue light intensity on the cell surface is 1 mW/cm^2^)^18^. The slide is then moved back to the storage rack, and the second (bottom) slide is delivered from the other storage rack to the assembly stage for the exposure of its photoalignment layer. At this point the printer’s worktable and the monomer mixture applicator are starting to heat up. The UV-curable glue mixed with spacers (calibrated glass balls, typically between 5 and 100 μm diameter, depending on the desired LCE film thickness) is applied in the four corners of the bottom slide. The top slide is placed onto the bottom one and the glue is cured by means of UV LED exposure for 10–50 s with the light intensity on the cell surface equal 18 mW/cm^2^. When the cell temperature stabilizes at 80 °C, it is filled with the liquid (molten) monomer mixture, transferred from its container on the heated dispenser tip (in several rounds, if needed for the cell to be completely filled by capillary forces). Finally, the worktable is cooled down and kept at 40 °C for 5 min to allow the liquid crystal monomer to orient and the mixture is polymerized by illumination with the same UV LED for 6 min. After the finished cell is transferred to the storage rack, RoboLEC is ready for the next cycle. The entire cycle takes around 4 h and 40 min, of which 4 h is taken up by the exposure of the photoalignment layers and about 30 min by heating and cooling of the worktable (compare Table 2 in the Supplementary Information for a detailed break-down of the cycle). The cells remain closed at the end of the cycle, as for some applications they may need to be opened under specific conditions or just before the next step of preparation.

To demonstrate RoboLEC’s capacity for automated LCE film fabrication with complex director patterns, we fabricated 50 μm thick films with twisted nematic orientation alternating between neighboring, 3 mm wide stripes. Previously such films, used to make light-driven actuators, have been prepared with manual assembly of glass cells with either photoalignment layers^19^ or patterned rubbing through masks^20,21^ and they have been used in millimeter-scale light-powered crawling robots^20^ and micromechanical devices, e.g. a linear stepping motor^21^. Inspection of the hand-made film with patterned alignment and the film of the same type fabricated with RoboLEC with polarized light microscopy shows that both films exhibit the desired molecular order (Fig. 1b,c) —sharp boundaries between areas with different orientation are visible and high contrast between them indicates high degree of molecular order. We have also tested the reproducibility of the process by running it a few times and comparing the resulting films (see Supplementary Information for details).

## Conclusions and outlook

Using a few of-the-shelf components, the most expensive being the 3D extrusion printer, we were able to build a robotic workstation for LCE film fabrication. As the glass slides used for the cells have to be spin-coated with the polyimide layer for photo-alignment, and the monomer mixture has to be prepared beforehand, the process can be considered semi-automated. In the current prototype, the racks were design to hold three plates for bottom and top of the cell. Within the same mechanical framework, this can be extended to ten, which would already allow for ten cells to be made without reloading, taking more than 40 h of continuous work. For even more cells, a separate glass plate feeder (e.g. carousel) can be added. The next generation of RoboLEC may also feature a rotating polarizer for photoalignment, thus opening up the possibility of fully automated preparation of LCE films with arbitrary 2.5 D director distribution^12^. This demonstrates how repeatable manual tasks—handling and assembling of small objects, fluid dispensing, image projection—can be automated with reasonable effort and expense. Thus, preparing a series of LCE films becomes possible with limited operator time needed to prepare the components (glass plates, glue with spacer(s), monomer mixture) and to set up the process. While the time required to prepare an LCE film remains the same as with the manual handling, the robotic system offers the capability of an unsupervised search for the optimum parameters—a procedure often involved in materials science—as it can produce several films with varying parameters, such as compound concentration, thickness, polymerization time, or temperature at different stages of the process and, with some modification, characterize their parameters, e.g. molecular orientation, with polarized light transmission measurements.

More broadly, our results show that automation of repeatable laboratory tasks is now within reach of even small research teams. This may evolve in two directions: one involves custom-made systems, designed for specific, unique applications, while the other, even more interesting, may offer flexible, modular systems that could integrate various units into automated workstations. Time will tell how these two branches will evolve and be adopted, both by the laboratory equipment manufacturers and by their potential users.

## Supplementary Information

Supplementary Information 1.

Supplementary Video 1.

## Data Availability

The datasets used and/or analyzed during the current study are available from the corresponding author on reasonable request.

## References

[1] Susskind D (2020). A World Without Work: Technology, Automation and How We Should Respond.

[2] du Sautoy M (2019). The creativity code Creativity and Fantasy.

[3] Blow N (2008). Lab automation: Tales along the road to automation. Nat. Methods.

[4] Ouyang W (2021). An open-source modular framework for automated pipetting and imaging applications. Adv. Biol..

[5] Burger B (2020). A mobile robotic chemist. Nature.

[6] Yu C, Xiong Q, Yang K, Chen H, Pan F (2021). A programmable and automated platform for integrated synthesis and evaluation of water electrolysis catalysts. Adv. Mater. Technol..

[7] Coley, C. W. *et al.* A robotic platform for flow synthesis of organic compounds informed by AI planning. *Science* 365 (2019). 10.1126/science.aax1566 31395756

[8] Steiner, S. *et al.* Organic synthesis in a modular robotic system driven by a chemical programming language. *Science* 363 (2019). 10.1126/science.aav2211 30498165

[9] Pilz da Cunha M, Debije MG, Schenning APHJ (2020). Bioinspired light-driven soft robots based on liquid crystal polymers. Chem. Soc. Rev..

[10] Qin L, Liu X, Yu Y (2021). Soft actuators of liquid crystal polymers fueled by light from ultraviolet to near infrared. Adv. Opt. Mater..

[11] McConney ME (2013). Topography from topology: Photoinduced surface features generated in liquid crystal polymer networks. Adv. Mater..

[12] Ware TH, McConney ME, Wie JJ, Tondiglia VP, White TJ (2015). Actuating materials Voxelated liquid crystal elastomers. Science.

[13] Chigrinov VG (2013). Photoaligning and photopatterning — a new challenge in liquid crystal photonics. Crystals.

[14] Iimura, Y., Akiyama, H., Li, X. T. & Kobayashi, S. Photoalignment control of LC and its applications to LCD fabrication. *in Liquid Crystal Materials, Devices, and Applications VI* 3297 (1998).

[15] Schadt M (2017). Liquid crystal displays, LC-materials and LPP photo-alignment. Mol. Cryst. Liq. Cryst..

[16] Miyachi, K., Kobayashi, K., Yamada, Y. & Mizushima, S. 41.1 : Distinguished paper: The world’s first photo alignment LCD technology applied to generation ten factory. *Dig. Tech. Papers* 41 (2010).

[17] Konieczkowska J, Kozanecka-Szmigiel A, Piecek W, Weglowski R, Schab-Balcerzak E (2018). Azopolyimides – influence of chemical structure on azochromophore photo-orientation efficiency. Polimery.

[18] Wani OM, Zeng H, Wasylczyk P, Priimagi A (2018). Programming photoresponse in liquid crystal polymer actuators with laser projector. Adv. Opt. Mater..

[19] de Haan LT (2014). Accordion-like actuators of multiple 3D patterned liquid crystal polymer films. Adv. Funct. Mater..

[20] Rogóż M, Zeng H, Xuan C, Wiersma DS, Wasylczyk P (2016). Light-driven soft robot mimics caterpillar locomotion in natural scale. Adv. Opt. Mater..

[21] Rogóż M, Haberko J, Wasylczyk P (2021). Light-driven linear inchworm motor based on liquid crystal elastomer actuators fabricated with rubbing overwriting. Materials.

