# Supplementary material for: Automated photo-aligned liquid crystal elastomer film fabrication with a low-tech, home-built robotic workstation
Source: Sci Rep. 2022 Oct 20;12:17598. doi: 10.1038/s41598-022-22556-8 (PMC9584969; doi:10.1038/s41598-022-22556-8)
Supplement: Supplementary file 1 — Supplementary Information 1. [file 41598_2022_22556_MOESM1_ESM.docx]

**Supplementary Information**

**Liquid crystal monomer mixture**

The monomer mixture contained 90 wt% of the monomer (4-methoxybenzoic acid 4-(6-acryloyloxy-hexyloxy) phenyl ester, Synthon), 8.5 wt% of the crosslinker (1,4-Bis-[4-(3-acryloyloxypropyloxy)benzoyloxy]-2-methylbenzene, Synthon), 1 wt% of the photoinitiator (2-benzyl-2-(dimethylamino)-4′-morpholinobutyrophenone, Irgacure 369, Sigma-Aldrich) and 0.5 wt% of the red dye (N-ethyl-N-(2-hydroxyethyl)-4-(4-nitrophenylazo)aniline, Disperse Red 1, Sigma-Aldrich). The compounds were molten and mixed on a hot plate at 140°C for 60 minutes.

**Table 1**

**List of components used to build the RoboLEC robotic workstation**

| **Item** | **Price (USD)** |
| --- | --- |
| **Flashforge Creator PRO 3D printer*** | **999** |
| Sony MP-CD1 laser computer projector** | 399 |
| Raspberry Pi 3 model B | 57 |
| Charger and USB A to microUSB connector and USB A to USB B connector | 13 |
| **Toshiba MicroSD 16 GB memory card*** | **4** |
| 1’’ dia., f= 50mm uncoated lens (Thorlabs LB1471) for the laser projector | 41 |
| ABS filament, 1.75 mm dia. | 9 (29 per kg) |
| UV LED and electronics for its integration with the 3D printer controls | 18 |
| Total cost*** | 1540 **(1003)** |

*Items **in bold** were bought specifically for the project. The other components were already available in our lab.

**The Sony MP-CD1 Projector was released in Apr 2018 and discontinued in Feb 2020.

***The total sum does not include a few machined metal parts (all of which have been made from scrap metal), nuts, bolts and wires.

**Table 2**

**Timing of the LCE film fabrication procedure**

| Moving the top slide to the assembly stage | 10s | Top slide preparation | 2h 00min 18s |
| --- | --- | --- | --- |
| Projecting the photoalignment pattern on the top slide | 2h 00min 3s |  |  |
| Moving the top glass slide back to storage rack | 5s |  |  |
| Moving the bottom slide to the assembly stage | 8s | Bottom slide preparation | 2h 00min 11s |
| Projecting the photoalignment pattern on the bottom slide | 2h 00min 3s |  |  |
| Switching on the heating of the worktable and of the liquid monomer applicator |  | | |
| Applying the glue - 1st corner | 7s | Application of the UV curable glue with spacers | 25s |
| Applying the glue - 2nd corner | 6s |  |  |
| Applying the glue - 3rd corner | 6s |  |  |
| Applying the glue - 4th corner | 6s |  |  |
| Moving the top slide onto the bottom slide | 22s | Assembling the cell | 38s |
| Curing the glue with UV LED (10s) | 16s |  |  |
| Waiting for temperature to reach 80°C and stabilise | ~7min | | |
| Applying the 1st portion of the liquid monomer mixture | 14s | Filling the cell with liquid monomer mixture | 1min 38s |
| Applying the 2nd portion of the liquid monomer mixture | 12s |  |  |
| Applying 3rd portion of the liquid monomer mixture | 1min 12s |  |  |
| Cooling down the worktable to 40°C | ~25min | | |
| LCE polymerization with the UV LED | 6min 2s | | |
| Moving the finished cell with the LCE film to the storage rack | 9s | | |
| **Total time** | **~4h 40min** | | |

**The reproducibility test of automated LCE film fabrication**

The glass slides have been prepared by spin-coating with a photoalignment Brilliant Yellow dye (BY) 0.5% and Polyvinylpyrrolidone (PVP) 0.1% solution in water, dried on a hot plate at 80 °C for 15 minutes. The process, repeated a few times to collect statistical data, starts with lifting the first (top) glass slide (21×38×1 mm³) from the storage rack and placing it onto the assembly stage. Here, the computer laser projector illuminates the glass slide through one polarizer with a white screen image for 10 minutes (light intensity on the glass surface is 3 mW/cm^2^) . The slide is then moved back to the storage rack, and the second (bottom) slide is delivered from the other storage rack to the assembly stage for the exposure of its photoalignment layer to black and white stripes pattern through one polarizer for 10 minutes and a complementary image of the stripes through a perpendicular polarizer for another 10 minutes. At this point heating up of the printer worktable and the monomer mixture applicator (the nib) begin. The UV-curable glue mixed with spacers (calibrated 50 μm diameter glass beads) is applied in the four corners of the bottom slide. The top slide is placed onto the bottom one and the glue is cured with a UV LED for 10 seconds (light intensity on the glass surface is 18 mW/cm^2^). When the cell temperature stabilizes at 80°C, it is filled with the liquid (molten) monomer mixture, transferred from its container via the heated dispenser tip in two rounds for the cell to be completely filled by capillary forces. Finally, the worktable is cooled down and kept at 40°C for 5 minutes to allow the liquid crystal monomer to orient and the mixture is polymerized by illumination with the same UV LED for 6 minutes. Finally, the finished cell with polymerized film is transferred to the storage rack.


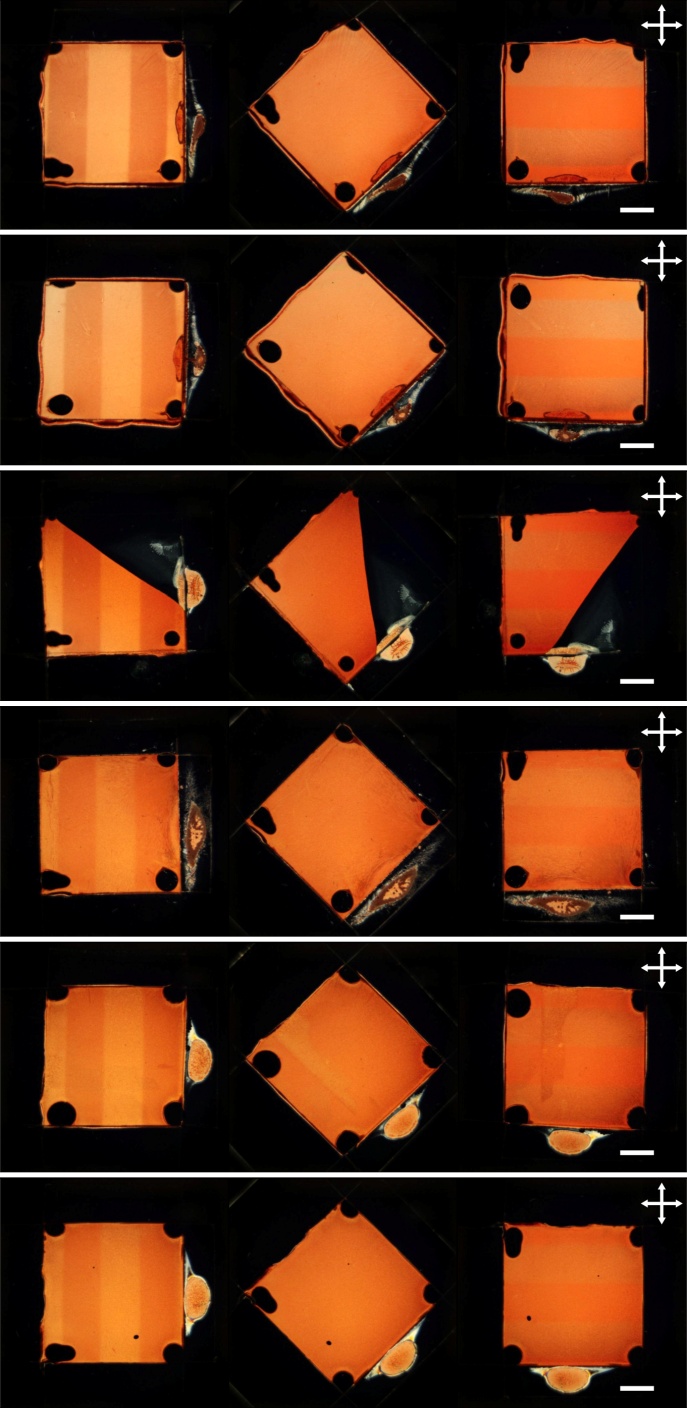


Figure SI 1. The reproducibility test results. Although there are visible faults in some of the LCE films (cell not filled completely due to the monomer mixture running out in run #3, an air bubble in run #6), these results are qualitatively comparable with the manual procedures and all resulted in an LCE film that could be useful for subsequent experiments. Many such faults might be eliminated with a simple camera and a basic image analysis software that would supervise the cell assembly and filling. Polarized white light photographs at three different orientations, the cross shows the polarizer orientation, the white scale bar is 5 mm long.
